# Stand-off nuclear reactor monitoring with neutron detectors for safeguards and non-proliferation applications

**DOI:** 10.1038/s41467-019-09967-4

**Published:** 2019-04-29

**Authors:** B. M. van der Ende, L. Li, D. Godin, B. Sur

**Affiliations:** Canadian Nuclear Laboratories, 286 Plant Road, Chalk River, ON K0J 1J0 Canada

**Keywords:** Nuclear fusion and fission, Experimental nuclear physics, Characterization and analytical techniques

## Abstract

Safeguards measures are employed at nuclear reactor facilities worldwide, to ensure that nuclear material is not diverted from peaceful uses. Typical safeguards measures involve periodic inspections, off-line verification and video surveillance of fuel cycle activities. Real-time verification of the fissile contents via stand-off monitoring can enhance continuity of knowledge for non-traditional reactor types, including research reactors and small modular reactors. Here we demonstrate the feasibility of using large-area neutron detectors for monitoring nuclear reactors at stand-off distances up to 100 m outside reactor shielding, as a potential reactor safeguards tool. Since the neutron yield per unit reactor power depends upon the isotopic composition of the reactor core, declared changes in fissile composition can be verified without accessing the core. The supporting results of experiments conducted at the National Research Universal reactor in Canada, are presented.

## Abstract (web summary)

Nuclear power reactors need to be monitored for safety and security while in operation. Here the authors discuss monitoring and safeguarding research reactors and small modular reactors using detection of neutrons up to a hundred meters away from the reactor shielding.

## Introduction

The International Atomic Energy Agency (IAEA) uses nuclear reactor safeguards measures to verify that nuclear material is not diverted from peaceful uses^1^. These measures are used to detect the diversion of declared nuclear materials, the misuse of declared nuclear facilities for undeclared nuclear material production, and the presence of undeclared nuclear facilities, materials, and activities anywhere in a state. Scenarios for diversion of nuclear material from peaceful uses can take a number of forms, such as misusing declared nuclear facilities for undeclared plutonium production, undeclared reductions in the level of irradiation of fuel to facilitate later removal of fissile material, or the actual undeclared diversion of fissile material from the reactor.

Typical safeguards measures at power reactors and research reactors worldwide often employ devices such as tamper-indicating seals (applied to reactor head, equipment hatches, transfer canals, and fuel assemblies), as well as unattended video surveillance cameras (applied to spent fuel pools). Safeguards inspectors also follow a number of procedures, including physical inventory verification of fresh fuel, seals, core fuel, spent fuel, and contents of containers/transfers; examination of accounting and operating records, and reports along with supporting documents; interim inspections to meet timeliness goals for detecting diversion of spent fuel, and verification of facility design information^2^. While the IAEA is relatively satisfied with its current safeguards approach at most power reactors, the IAEA sees higher priorities for its limited safeguards budget, including the monitoring of non-traditional reactor types, such as small modular reactors (SMRs) and research reactors^2–4^. Research reactors often classify as SMRs by definition^5^, and there are approximately 170 research reactors and critical assemblies under IAEA safeguards worldwide^3^. In contrast to power reactors, research reactors present significant safeguards concerns with their wide variation in design and operation, the type of fuel that is employed (about half of research reactors around the world employ highly-enriched uranium^6^), the reactor power and cooling capacity, significant potential for target irradiation, and the presence of and ease of access to hot cells. Sealed core SMRs in general also present further challenges to IAEA safeguards, as they preclude direct sampling of fuel at regular intervals for physical inventory verification^7^.

The IAEA has stated in its 2012–2023 Long-Term R&D Plan for its Department of Safeguards that it is a high priority to “develop instruments and associated techniques to detect the establishment of nuclear fuel cycle activities, for example by detecting process emanations”^4^. Such technologies can provide real-time additional information for verification of nuclear reactor fuel cycle activities, including cores of research reactors and other SMRs. A prominent emanation from fission nuclear reactors are neutrons.

In the following, the feasibility of neutron detection for monitoring reactor fuel cycle activities is examined, at significant stand-off distances from a nuclear reactor. To date, there has been published work about using neutron detectors outside of reactor shielding for determining power density distribution within reactors, from escaping fast neutrons^8^. Although the preceding publication may anticipate this work, this work explicitly demonstrates how escaping fast neutrons detected as thermalized neutrons can be related to monitoring the fissile isotope inventory of a reactor core, for safeguards purposes; in particular, the stand-off neutron count rate per unit reactor power is proportional to a weighted sum of the fissile isotope inventory. Further, it is shown in this work that neutron detection at stand-off distances outside of reactor shielding using an array of large-area neutron detectors, each weighing ~10 kg, at various locations at a reactor facility provides a viable, economical, and compact means of monitoring reactor neutron emanations. The economical aspect of using neutron detectors for the purpose of reactor safeguards means that it should be feasible to employ an array of detectors at different locations around a reactor, with coordinated detection signals such as to discriminate against either inadvertent or malicious interferences that might cause variations in individual neutron detection rates.

## Results

### Theory

The technique of stand-off reactor monitoring using neutron detection is based upon the fact that the number of neutrons detected *n*_det_, is proportional to the population of neutrons *n*_pop_ in the reactor core,

1 $$n_{{\mathrm{det}}} \propto n_{{\mathrm{pop}}} \propto \frac{{\left\langle \phi \right\rangle }}{{\left\langle v \right\rangle }}V,$$

where <*ϕ*>[n cm^−2^ s^−1^] is the average neutron flux in the reactor core, <*v*> [cm s^−1^] is the average speed of the neutrons in the reactor core, and *V* [cm^3^] is the volume of the reactor core. For monoenergetic incident neutrons, the volumetric rate of fission is given by ${\mathrm{\Sigma }}_{\mathrm{f}}\phi$, where ${\mathrm{\Sigma }}_{\mathrm{f}} = N\sigma _{\mathrm{f}}$ is the macroscopic fission cross-section, *σ*_f_ [cm^2^] is the microscopic cross-section, *ϕ* is the flux of monoenergetic neutrons, and *N* [atom cm^−3^] is the number density of the fissile nuclei. In a reactor, the neutron energy spectrum is not monoenergetic, the neutron flux varies with spatial location and time, and the spatial distribution of fissile material is not uniform, the fission rate is determined by integrating ${\mathrm{\Sigma }}_{\mathrm{f}}({\mathbf{r}},E_{\mathrm{n}},t)\phi ({\mathbf{r}},E_{\mathrm{n}},t)$ over all locations (**r**), and neutron energies (*E*_n_) in the reactor. For thermal neutron reactors where most fissions occur in the thermal neutron energy range, one may assume that *ϕ* is an appropriate space and energy average flux of thermal neutrons, and ${\mathrm{\Sigma }}_{\mathrm{f}}$ is a corresponding average macroscopic cross-section^9^. By multiplying ${\mathrm{\Sigma }}_{\mathrm{f}}\phi$ by the volume *V* of the reactor, as well as the energy released per fission $E_{\mathrm{f}}$, and further accounting for multiple fissile isotopic species being present in the reactor, the reactor thermal power $P_{{\mathrm{tot}}}$ may be estimated by

2 $$P_{{\mathrm{tot}}} = V\left\langle \phi \right\rangle \mathop {\sum }\limits_i \left\langle {{\mathrm{\Sigma }}_{{\mathrm{f}},i}} \right\rangle E_{{\mathrm{f}},i} = V\left\langle \phi \right\rangle \mathop {\sum }\limits_i N_i\left\langle {\sigma _{{\mathrm{f}},i}} \right\rangle E_{{\mathrm{f}},i},$$

where the summation index *i* runs over the fissile isotope species in the reactor core. The averaged components of Equation (2) should technically be integrals over energy and space, and therefore vary as a function of fuel burn-up distribution or refueling over time, but can be approximated above as appropriately averaged factors that do not vary with energy or as a function of location. Remarkably, as is demonstrated in this work, the average factors as written work very well under the assumptions stated and can therefore be used to verify the change in isotopic composition, *N*_*i*_, over time.

As can be seen from Equation (2), the technique is sensitive to isotopic differences in the energy released per fission, and the cross-section for fission^9^. Such properties for U-235, U-238, Pu-239, and Pu-241 are summarized in Table 1. While there are numerous actinides that contribute to neutron production in a nuclear reactor, these four isotopes account for 99.9% of the power in power reactors^10^. As shown in this work, these isotopic-dependent properties enable this technique to detect changes in isotopic composition of a reactor core, while monitoring the power and escaping neutron output of the reactor at stand-off distances of up to 100 m; it is remarkable that this can be achieved even with the simplifying assumptions made in Equation (2). As will be demonstrated in this work, essentially the varying fission cross-section and energy released per fission for each isotope means that the power output per circulating neutron varies according to the fissioning parent isotope, resulting in varying neutron flux per unit reactor power as the fissile isotope inventory varies in the reactor core.

**Table 1:** Properties of key reactor isotopes

Property	U-235	U-238	Pu-239	Pu-241
Thermal (0.0253 eV) neutron induced fission cross-section^20^	582.6 ± 1.1 barns	0.000003 barns	748.1 ± 2.0 barns	1011.1 ± 6.2 barns
Thermal energy per fission^21^	201.7 ± 0.6 MeV	205.0 ± 0.9 MeV	210.0 ± 0.9 MeV	212.4 ± 1.0 MeV

Equation (2) relies upon the energy released per fission, as well as the cross-section for fission, for each isotope. These quantities are presented above for isotopes which typically account for 99.9% of the power in a reactor core: U-235, U-238, Pu-239, and Pu-241^10^

### Detector locations

**Fig. 1:**
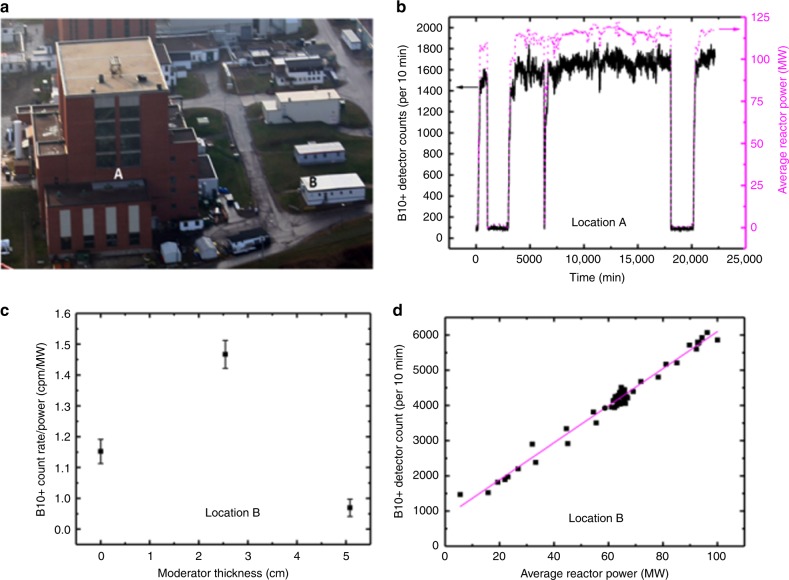
Detector count rate variations with reactor power, time, and moderator thickness. **a** Aerial view of the National Research Universal (NRU) reactor building (image copyright ©Canadian Nuclear Laboratories), inside of which was positioned Location A, 17 m from, and 2 levels below, the reactor core, while Location B was situated at ground level in a trailer outside of the NRU reactor building, 69 m from the reactor core. **b** B10+ detector raw count rate (solid black curve) and reactor thermal power averaged over 10 min (dotted magenta curve) versus time at Location A. **c** Variations in B10+ detector raw count rate (using a single detector tube) at Location B with high-density polyethylene (HDPE) moderator thickness. Error bars are standard deviations of the mean. **d** Measured B10+ neutron detector raw count rate at Location B versus reactor thermal power averaged over 10 min during reactor start-up and shut-down periods, with a linear regression fit to demonstrate their linear relationship. No corrections have been made for changing environmental factors

In this work, two large-area (18 × 100 cm^2^ detection area) boron-lined neutron detectors (heretofore referred to as BCS and B10+ detectors – see Methods section for details) were placed at two locations in proximity to a nuclear research reactor, the National Research Universal (NRU) reactor in Chalk River, Ontario, Canada (see Methods section for further description). One location (Location A) was within the NRU reactor building, placed approximately 17 m from the NRU reactor core, two levels below the main reactor floor. The other location (Location B) was outside of the NRU reactor building in a portable trailer building, approximately 69 m from the NRU reactor core. These locations are pictured in Fig. 1a, and were chosen for their difference in proximity to the reactor. It turns out that these locations also present significantly different shielding burden to neutrons incident on the detectors, as summarized in Table 2. For each neutron detector, its count rate as a function of time was recorded during the course of the measurement. These data are compared against NRU’s simultaneously measured thermal reactor power, as illustrated in Fig. 1b. During the course of neutron measurements, the NRU reactor typically underwent a scheduled shut-down and subsequent start-up every few weeks of operation. This provided an opportunity to test the sensitivity of the neutron detectors to reactor shut-down and start-up. Figure 1b shows that the neutron detector count rate correlated very well with NRU’s thermal reactor power, particularly during the course of reactor start-up and shut-down. Some background count rate due to neutrons from cosmic ray background radiation can be seen when the reactor was shutdown. Note that the signal (from NRU operating) to background (when NRU is shutdown) ratio for the B10+ detector was typically greater than 10:1 at locations A and B. The signal from Location A was significantly less in comparison to signal from Location B (by a factor of 2–4), despite Location A being at a closer distance to the reactor. This can be attributed to the difference in shielding and overburdening noted in Table 2 for the two locations.

**Table 2:** Intervening shielding/building material between the NRU reactor core and Locations (a) A and (b) B

a	
Material Type	Estimated thicknesses of material between Location A and NRU reactor core (cm)
Water	45
Steel	45
High-density concrete	700

b			
Material type	Estimated thicknesses of material between location b and nru reactor core (cm) main reactor hall top reactor shielding	Side reactor shielding	Reactor building exterior wall
Water	300	30	–
Steel	120	30	–
High-Density Concrete	–	270	15

a indicates that Location A was two floors below the NRU reactor core, within the reactor building

b indicates that Location B was at ground level outside of the reactor building. The main reactor hall was at ground level, enclosed by the reactor building exterior walls. The main reactor hall surrounded the top and side shielding of the reactor core

### Neutron moderation

The large area neutron detectors employed in this study were thermal neutron detectors; they were most sensitive in detecting neutrons in thermal equilibrium with their environment. Enhancement of their detection rate when exposed to neutrons with energy greater than that of thermal equilibrium can be achieved through the use of hydrogenous moderating material surrounding the detector. To determine qualitatively the average energy of the neutrons incident upon the neutron detectors, the thickness of high-density polyethylene (HDPE) surrounding a single detector tube at Location B was varied. Figure 1c shows how the detector count rate varied with HDPE moderator thickness, where zero thickness corresponded to no moderator surrounding the detector.

Previous work (data not shown) has demonstrated that 5 cm of HDPE is optimal for thermalizing neutrons of 2 MeV average energy from spontaneous fission of Cf-252. Figure 1c shows that 2.5 cm - thick HDPE was optimal for the detector count rate, suggesting that the average energy of neutrons incident on the detector was substantially <2 MeV, but greater than thermal energy, i.e., in an epithermal regime. This can be understood from the point of view that the neutron energy spectrum in a thermal, moderated reactor core contains a thermal spectral component peaked near thermal equilibrium energy, and a fission spectral component with average energy near 2 MeV;^11^ these spectral components are bridged by an epithermal regime. Absorption of neutrons within the reactor core preferentially selects low energy neutrons, due to their higher absorption cross-section relative to higher energy neutrons. The higher energy neutrons within the reactor correspondingly have a higher leakage probability. Figure 1c is consistent with the detector on average receiving higher energy neutrons that have been partially moderated by the reactor reflector, reactor shielding, and the detector’s exterior environment.

### Neutron count rate variation with reactor power

Figure 1b also shows that the neutron detection count rate followed the reactor thermal power through its temporal fluctuations well while NRU was at power. This suggests that the ratio of reactor power to detector count rate is a meaningful quantity to follow. Figure 1d further demonstrates how the neutron detection count rate had a clear linear dependence on nuclear reactor power. Here, a linear regression fit was applied to the B10+ detector raw count rate at Location B as a function of average reactor power, during reactor start-up and shut-down periods. It should be noted that the neutron detector count rate shown was *not* corrected for any environmental influences, whether from changing atmospheric conditions or changing operational environment; the contribution of these factors is in the minimal scatter present in the figure. It is clear, therefore, that neutron detection outside of reactor shielding can be used to monitor changes in the in-core neutron flux, which is a useful capability for monitoring and verifying nuclear reactor fuel cycle activities, from a nuclear safeguards point of view. While neutron detection outside reactor shielding has been used to monitor reactor power in the past, the current application demonstrates the capability to do so at stand-off distances.

### Neutron count rate variation with isotopic inventory

The attribute that makes neutron monitoring outside of reactor shielding a useful tool for verification purposes in safeguards applications, is that the technique is sensitive to changes in fissile isotope inventory in the reactor core. To see this, one can re-arrange Equation (2) into the following expression,

3 $$\frac{{\left\langle \phi \right\rangle }}{{P_{{\mathrm{tot}}}}} = \left[ {N_{\mathrm{A}}\mathop {\sum }\limits_i \frac{{m_i\left\langle {\sigma _{{\mathrm{f}},i}} \right\rangle E_{{\mathrm{f}},i}}}{{w_i}}} \right]^{ - 1},$$

where the number density *N*_*i*_ for the *i*^th^ fissile isotope and the volume *V* of the reactor core are re-written in terms of Avogadro’s number *N*_A_ [atom mol^-1^], the mass *m*_*i*_ [g] of the *i*^th^ fissile isotope, and corresponding atomic weight *w*_*i*_ [g mol^-1^]. Consistent with Equation (1), the neutron detector count rate per unit reactor power is assumed to be proportional to <*ϕ>*/*P*_tot_. Equation (3) shows a clear linear weighting dependence of the contribution of each fissile isotope to the average in-core neutron flux per unit thermal reactor power.

In our work, the neutron detector count rate was recorded as a function of time, over the course of weeks and even months. In the simplified model above, the quantities that varied in Eq. (3) over this time scale were the fissile isotope masses *m*_*i*_, particularly as the U-235 in fresh fuel was burned up and other isotopes of U and Pu were produced via transmutation or introduced via on-line refueling. The masses of the isotopes present within the NRU reactor were carefully followed with a three-dimensional neutron diffusion code in two energy groups, known as TRIAD^12^. As the NRU reactor underwent online re-fueling on a routine basis, the fissile isotope inventory of the reactor core normally remained relatively constant. However, the inventory of fuel in the reactor core varied with time as, for instance, the quantity of Co-59 absorber in the core was varied for production of high specific activity Co-60. Changes such as these impact the frequency of re-fueling, and by consequence, the maintained fissile uranium and plutonium mass inventory in the core changed significantly.

**Fig. 2:**
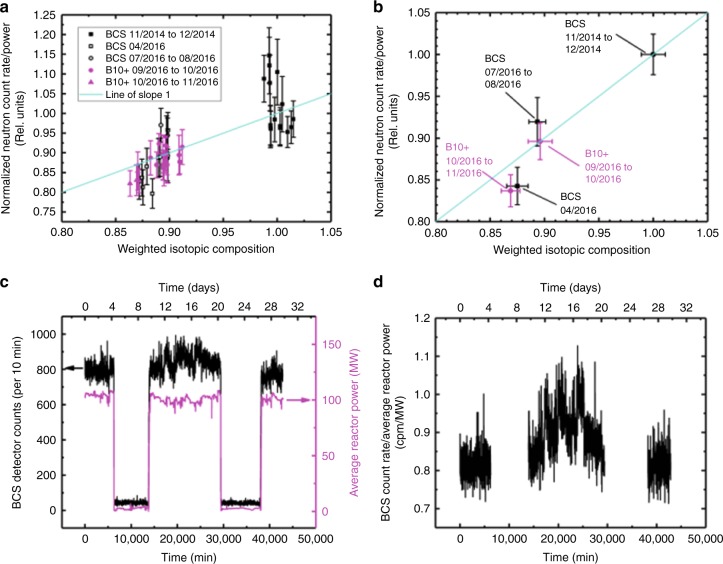
Dependence of neutron detector count rate on reactor power and isotopic inventory. **a** Relative measured neutron detector count rate (with background subtracted) per unit reactor power (ordinate) vs. weighted isotopic composition of the reactor core (abscissa). The abscissa values are determined by individual TRIAD simulation core followings during particular periods of time as indicated in the graph legend. The ordinate values are determined by neutron measurements conducted with either the boron-coated straw (BCS) detector or the B10+detector at the time corresponding to each TRIAD core following. All quantities here are presented relative to a reference case, chosen as the average of data from the time period 2014/11 to 2014/12. Ordinate error bars are standard deviations of the normalized neutron count rates per unit power measured over the time period between each TRIAD core following calculation. The data points are plotted against a line of slope 1, with zero intercept. **b** Data from (**a**), averaged together in 1-month-long periods, as indicated in the graph. Data points in black squares were recorded at Location A with the BCS detector, and data points in magenta circles were recorded at Location B with the B10+ detector. These points collectively are seen to coincide well with a line of slope 1, and zero intercept. The error bars are standard deviations of the mean of data points from (**a**) for each time period. **c** Representative BCS detector count rate (at location A, from 2014/11 to 2014/12 time period) versus time, including periods of reactor start-up and shutdown. **d** Data from (**c**), relative to reactor power, and excluding shutdown periods, to illustrate how the detector count rate per unit reactor power can vary over the course of a month

Since there was no absolute calibration available, the change in fissile inventory was tracked by comparing the neutron count rate per unit power for the case of interest to a base reference case. Figure 2a shows how the measured neutron detector net count rate (background subtracted) per unit reactor power varied with weighted fissile isotopic composition where both quantities are shown as a ratio with respect to a reference case (i.e., the reference case has value 1). The weighted fissile isotopic composition was based on fissile isotope mass inventories present at the time of measurement, estimated from TRIAD simulations. This graph shows the average neutron count rate per unit power at the time of each TRIAD core following calculation. Figure 2b shows the same data, but averaged over month-long time periods. The primary reference case in Fig. 2a, b is data for the earliest (2014/11 to 2014/12) time period, taken with the BCS detector. Some datasets were taken with the B10+ detector, whose detection efficiency differs substantially from the BCS detector; the neutron count rate per unit power for B10+ cases are presented relative to the average neutron count rate per unit power for the earliest B10+ case (Sep 2016 to Oct 2016 time period), while normalizing the weighted isotopic composition relative to the (Nov 2014 to Dec 2014) case. Equations (1–3) predict that there should be a one-to-one correspondence between the neutron count rate per unit reactor power, and the weighted isotopic composition, and this is borne out in how close the data points lie to a line of slope 1, and zero intercept. Indeed, linear fitting on data in Fig. 2a yields a slope of 1.08 ± 0.13 (in agreement with slope 1, within standard error), and an ordinate intercept of −0.08 ± 0.12 (close to zero). The ordinate error bars in Fig. 2a are standard deviations of the normalized neutron count rates per unit power measured over the time period between each TRIAD core following calculation. These error bars take into account the variations caused by environmental and operational factors, and are much larger than the standard errors from counting statistics alone. The ordinate error bars Fig. 2b are standard deviations of the mean of data points from Fig. 2a, over each monthly time period. The abscissa error bars in Fig. 2b account for the variations in the TRIAD core following quantities over the course of each monthly period.

Table 3 displays the average absolute masses of the fissile isotopes U-235, Pu-239, and Pu-241 during the month-long time periods shown in Fig. 2a, b. These are the fissile isotopes which contributed greater than 90% of the average neutron flux per unit power in the reactor core. A small percentage also comes from fission of U-238; changes in U-238 mass are included in Table 3 for reference. Using Equation (3), the three primary fissile isotope masses can be used to compute the relative neutron flux per unit power in the reactor, which are seen to agree with average relative measured detector count rate per unit power for the corresponding time period, within 5%. It can be seen in Table 3 that the changes in fissile isotope inventory were dominated by simultaneous changes in U-235 and Pu-239 content. For comparison, a 1 kg change in U-235 inventory alone is estimated to produce a 4% change in the neutron flux per unit reactor power, while a 100 g change in Pu-239 inventory alone is estimated to produce a 0.5% change in the neutron flux per unit reactor power.

**Table 3:** Average masses of fissile isotopes for the time periods indicated in Fig. 2a, b

a							
Time Period	U-235 (g)	Pu-239 (g)	U-238 (g)	Pu-241 (g)	Relative calculated <*ϕ*>/*P*_tot_	Relative measured count rate/power	Percentage difference (%) [|meas − calc| / (meas + calc) / 2]
11/2014 to 12/2014	23045.49	434.74	182879.33	32.20	1	1	0.0
04/2016	+ 3223.14 (13.98%)	+ 36.55 (8.41%)	+ 73842.34 (40.38%)	-11.06 (-34.35%)	0.879	0.843	4.3
07/2016 to 08/2016	+ 2634.01 (11.43%)	+ 107.11 (24.64%)	+ 77463.17 (42.36%)	-8.77 (-27.24%)	0.895	0.920	2.7

b							
Time Period	U-235 (g)	Pu-239 (g)	U-238 (g)	Pu-241 (g)	Relative calculated < $\phi$ > /*P*_tot_	Relative measured count rate/power	Percentage difference (%) [|meas – calc | /(meas + calc) / 2]
09/2016 to 10/2016	+ 1857.57 (8.06%)	+ 620.66 (142.76%)	+ 290566.29 (158.88%)	+ 41.98 (130.37%)	0.896	0.896	0.0
10/2016 to 11/2016	+ 2386.76 (10.36%)	+ 793.51 (182.53%)	+ 277483.17 (151.73%)	+ 80.94 (251.37%)	0.868	0.837	3.6

Using Eq. (3), the relative neutron flux per unit reactor power is estimated from masses of fissile isotopes which contribute greater than 99.9% of the neutron flux in the reactor core. The estimated relative neutron flux is compared against the average relative detector count rate per unit reactor power for the corresponding time period, measured with (**a**) the BCS detector at location A, and (**b**) the B10 + detector at location B. All changes in mass amounts (and corresponding percentage changes indicated in parentheses) are relative to the 2014/11 to 2014/12 time period

**Table 4:** Change in weighted isotopic composition from removal of 1 kg of U-235 or Pu-239

Reactor	Change in weighted isotopic composition for removal of 1 kg from mass inventory of		Relative change in U-235+Pu-239+Pu-241 mass (%)
	U-235	Pu-239	
NRU	0.0393	0.0523	3.84
Molten salt reactor (MSR)	0.00962	0.0127	0.911
High temperature gas reactor (HTGR)	0.00609	0.00802	0.570

The change in weighted isotopic composition is compared against the corresponding relative change in total sum of U-235, Pu-239, and Pu-241 fissile mass inventories. The MSR and HTGR cases are computed after 1 year of burn-up; the NRU case is computed from data during the 2016/09 to 2016/10 time period

## Discussion

The results demonstrate that the in-core neutron flux per unit reactor power, and the resulting neutron count rate outside of reactor shielding, vary as the relative number of atoms of the fissile isotopes present within the reactor core; as shown in Eqs. (2) and (3). Each fissile isotope will provide a weighted contribution to the neutron flux per unit power: calculated from Eq. (2) using data shown in Table 1, the contribution is expected to be 1.337 ± 0.009 times larger for Pu-239 than for U-235, simply on the basis of considering their differences in fission cross-section and energy released per fission.

It is remarkable that Equation (3), being a relatively simple equation based on significant assumptions and simplifications described earlier in this article, is found to be in good agreement with our experimental observations, and with simple considerations based on fission cross-section and energy released per fission for differing isotopic species. This demonstrates that one can track or verify changes in the total amount of fissile content in the core of a research reactor such as NRU, and, by extension for thermal nuclear reactors with sealed cores (such as some proposed small modular reactor designs), or continuous refueling (such as pressurized heavy water reactors), or other light-water reactors. Due to the proportionality between the number of fissile atoms present in the reactor core, and the neutron flux per unit power ratio, the accuracy with which one can detect or verify a fractional change in fissile isotope content is the same accuracy with which one can measure the neutron flux to power ratio. As shown in this work this accuracy is limited in practice by variations caused by environmental and operational factors and not by counting statistics. It follows that the verification of isotopic content with the above methods will likely benefit from improved neutron transport simulations and by implementation of data analytics techniques.

Figures 1d, 2a, and 2b show that the accuracy of the neutron to power ratio in the present test case is limited to <5% due to environmental and operational variations and not by statistical uncertainty in the count rate. Therefore, any change in fissile inventory that causes an approximately 5% change in the neutron to power ratio (i.e., a change of ~1 kg of U-235 or Pu-239) can be measured at NRU within a time period required to obtain comparable statistical accuracy (i.e., approximately 400 counts) for neutron counts and for power measurement.

In the present practical demonstration, the data shown in Fig. 2b show that average neutron count rate for 2014/11 to 2014/12 data is separated from the average neutron count rate for 2016/04 data by more than 4 standard deviations of the mean–they are distinct from each other with more than 95% confidence. Since each data point is based on averaging over at most 4 weeks, this is established within 8 weeks of data, and corresponds to addition of 3.22 kg of U-235 with 0.036 kg Pu-239 (Table 3), a sum total of 3.26 kg fissile isotope mass. Movement (whether added to or diverted from the core) of 3.26 kg of Pu-239 alone would be detected with similar sensitivity and timeliness. For comparison, the IAEA’s safeguards timeliness goal for detecting diversion of a significant quantity of Pu (8 kg) from spent fuel in a civil nuclear facility is 90 days^13^. The data shown in Table 3 therefore demonstrates that with neutron detection at stand-off distances outside of a reactor core, it is possible to detect the movement of kilogram quantities of fissile Pu and U isotopes within 90 days.

**Fig. 3:**
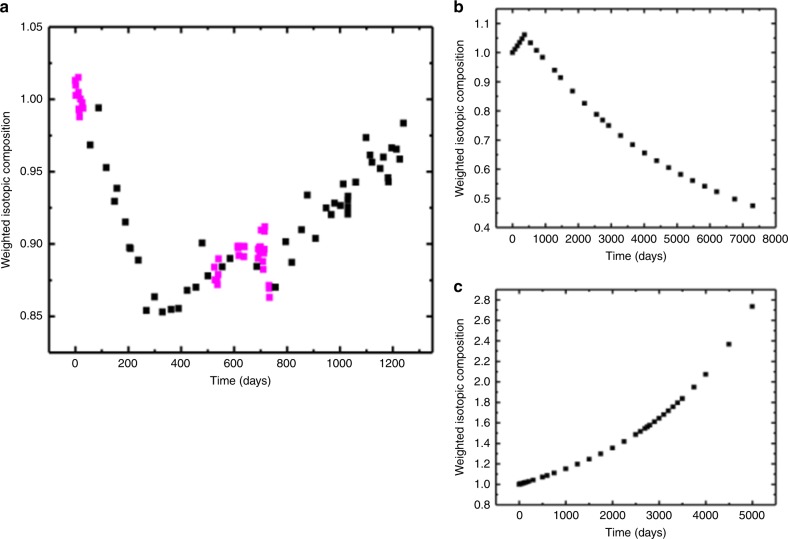
Weighted isotopic composition of small modular reactor cores versus time. **a** Weighted isotopic composition of the NRU core versus time, based upon data from core-following software. Points in magenta indicate times for which neutron count rate data presented in this work was taken. **b** Weighted isotopic composition of a simulated 3021 kg 4% LEU molten salt reactor core, versus time^14^. **c** Weighted isotopic composition of a simulated 956 kg 19.8% LEU high-temperature gas-cooled reactor core, versus time^14^

While the above has been demonstrated for the NRU research reactor, Fig. 3 shows how this can work for other types of reactors. Figure 3a displays the weighted isotopic composition of the core of NRU versus time, in comparison with two types of small modular concept reactors: Fig. 3b displays a molten salt reactor (MSR), and Fig. 3c displays a high temperature gas-cooled reactor (HTGR). While data in Fig. 3a are based on data from TRIAD, results in Fig. 3b, c are calculated from results of previously published simulations of concept cores (S. Golesorkhi, personal communication)^14^. Each SMR concept was modeled in long-term operation without manual intervention. It is quite evident that the weighted isotopic composition as calculated in Equation (3) can vary significantly with time in each case, and provides a baseline against which anomalies due to fuel diversion or misuse scenarios during a typical fuel cycle could be detected. In the case of the MSR simulation, automated online addition of U-235-bearing fresh fuel to counteract fission products leads to a rise in U-235 mass in the core, and a corresponding drop in the weighted isotopic composition. In the case of the HTGR simulation, burn-up of U-235 and build-up of plutonium isotopes leads to a non-linear monotonic increase in the weighted isotopic composition.

Table 4 summarizes the change in weighted isotopic composition that occurs in each reactor core with the removal of either 1 kg of U-235 or 1 kg of Pu-239; the size of this change is seen to scale with the corresponding percentage change that occurs in the sum of the U-235 and Pu-239 mass inventory (the dominant contributors to neutrons in the core from fission) with this 1-kg removal. The mechanism for monitoring the variation in isotopic composition with stand-off neutron detectors, by detecting leakage neutrons from the core that are proportional to the population of neutrons in the reactor core (see Equation (1)), is the same for each reactor type. Hence, the magnitude of the change in monitoring neutron detection rate will also scale with the percentage change that occurs in the sum of the U-235, Pu-239, and Pu-241 mass inventories.

It should be stressed that the sensitivity of the stand-off neutron monitoring method hinges on the detection efficiency of the neutron detector that is employed, and the environment in which the detector is placed. The environment includes the size of the reactor and the neutron flux that it produces, the nature and extent of the reactor shielding and the neutron flux leakage that it permits, the overburden/influence of other building infrastructure that may exist between the exterior of the reactor shielding and the neutron detector’s location, and the changing operational environment. While one should be aware of the sensitivity to the operational environment to be a potential source of interference to monitoring the reactor core, the potential for strategically located neutron detectors to exploit their sensitivity to local environment for monitoring fuel movements outside of the reactor core should also be noted.

**Fig. 4:**
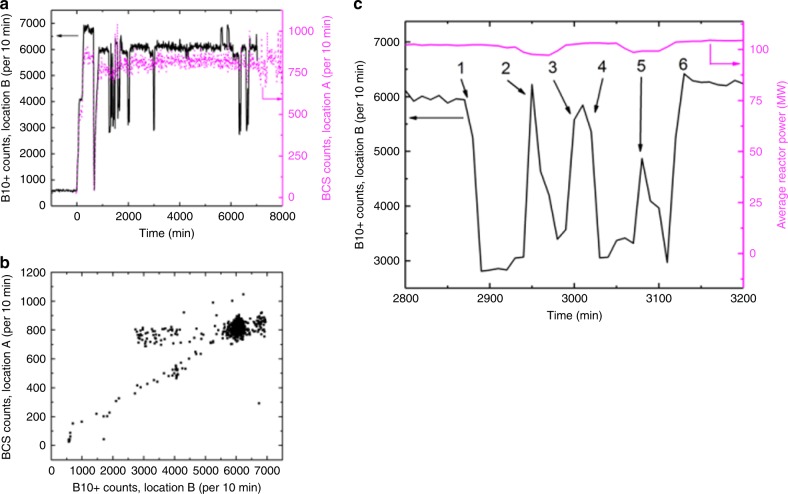
Detector sensitivity to local environment. **a** B10+ detector count rate at Location B, and simultaneous BCS detector count rate at Location A, versus time. **b** The same data from (**a**), demonstrating the correlation between the B10+ and BCS count rates. **c** An example of how the B10+ detector count rate varied with online refueling activities: (1) fuel rod flask parked on top of reactor, (2) fuel rod inserted into reactor core from flask at one rod location, followed by removing a fuel rod from reactor core into flask at another rod location, (3) flask moved away from top of reactor for a break, (4), flask moved back onto top of reactor, (5) fuel rod inserted into reactor core from flask at one rod location, followed by removing a fuel rod from reactor core into flask at another rod location, (6) flask moved away from top of reactor

To give an idea of the influence of the local environment, Fig. 4 compares BCS detector data acquired at Location A near NRU with B10+ detector data taken simultaneously at Location B. From Fig. 4a, it is evident that the B10+ detector count-rate at Location B was 7.5 times greater on average than the BCS detector count-rate at Location A. Although the B10+ detector was innately more efficient at detecting neutrons than the BCS detector by nearly a factor of 2, Location A also presented substantially more environmental shielding and overburden than Location B, as summarized in Table 2.

Figure 4a also shows that Location B data exhibited regular 50% decreases in detector signal, while the Location A data did not. Figure 4b shows that in spite of these regular 50% deviations in signal at Location B, the simultaneous signals from Locations A and B correlated very well with each other. In fact, the 50% deviations in detection rate at Location B match the timing of online refueling activities occurring at the top of the reactor, as detailed in Fig. 4c. Although the reactor power sometimes decreased during online refueling, it did not always do so, and the B10+ count rate dropped well before there was any change in reactor power, if there was any. Rather, the drop in B10+ count rate resulted from the fuel rod flask blocking neutrons escaping from the top of the reactor, when the flask was positioned over top of the reactor during reactor rod movement. The spikes in count rate often observed in the middle of the drop in B10+ count rate coincided with an exchange of fuel rods between the reactor core and the flask. As seen in Fig. 4b, these events were not recorded at Location A: the BCS detector at Location A maintained a count rate between 70 and 80 counts per minute while the B10+ detector at Location B occasionally recorded a ~50% drop in count rate during these events. It is likely that the position of Location A and the significant amount of fixed shielding presented to this location prevented the BCS detector at Location A from seeing neutrons from the top of the reactor.

The above discussion highlights the importance of the local environment to the stand-off monitoring detector, seeing how a detector at Location B could detect online refueling events, while a detector at Location A could not. The transitory interferences recorded at Location B could be properly distinguished only with the data from Location A where such interference did not occur. This speaks to the importance of placing multiple detectors at multiple locations around a reactor, and coordinating their signals together, in order to discriminate against interfering detector signals. Environmental factors together with the capability of the neutron detectors to be employed must be carefully evaluated to ensure that IAEA safeguards goals can be met. This evaluation would include careful time-based list mode data monitoring of normal reactor activities with the neutron detector in its intended location, in order to establish a reliable reference baseline. Optimization of the reference baseline can be achieved through improving the detector efficiency (by either selecting more efficient detection technology strategies, or larger detection area), and determining optimal locations for detecting neutrons with the highest signal-to-noise ratio possible. Anomalous activities within the reactor core can then be seen through statistically significant departures from this baseline, as a function of time.

Examples of interference of neutron detection count rate with malicious intent include the following: (a) placing more moderator around each detector, sufficient to maintain a constant count rate as the reactor is set to operate at a higher power level, or (b) placing a neutron source in front of each detector to emulate the expected neutron signature while an adversary shuts down the reactor for the purposes of diverting fuel from the reactor core. In each case, where one possesses a good baseline of how each neutron detector in a coordinated network is expected to behave as a function of time (with, say, 10 minute time resolution or less) in its local environment, it would be quite challenging to maintain the expected neutron behaviour at all instances in time, at all detector locations: significant anomalous behaviour in any of the detector readings would present an occasion for further investigation. Assuming, however, that each scenario is accomplished without detectable anomalous behaviour, one can use other safeguards measures to mitigate/overcome this shortcoming. Design information verification in the form of random in-person inspections to check the detector calibration against the declared facility design, for example, could pick up the presence of additional moderator shielding, or neutron sources placed in front of the detectors.

On the other hand, the clarity with which the B10+ detector at Location B was able to record the online refueling events shows the immense potential of using neutron detectors at stand-off locations for non-invasively tracking reactor facility operations, for safeguards purposes.

In summary, this work has demonstrated that large-area neutron detectors can be employed for monitoring nuclear reactor power and isotopic inventory at stand-off distances of up to approximately 100 m from the NRU research reactor. Preliminary studies (data not shown) indicate that neutron monitoring can also be conducted at stand-off distances outside of containment of commercial pressurized heavy water reactors. It should be noted however, that the technique of neutron monitoring can also be implemented within containment using, for example, existing neutron detectors for criticality alarms. A coordinated network of neutron detectors dedicated to reactor monitoring can provide an economical and practical means of supporting the achievement of IAEA safeguards goals through detecting process emanations from the establishment of nuclear fuel cycle activities.

## Methods

### Nuclear reactor

Large-area neutron detectors have been used for detection of neutrons from operation of the National Research Universal (NRU) nuclear reactor, at stand-off distances. The NRU reactor at Chalk River Laboratories was used to carry out research in basic science and in support of the Canadian nuclear power programs. It was also a major global supplier of medical radioisotopes. The NRU reactor was heavy water cooled and moderated, with online re-fueling capability. It was licensed to operate at a maximum power of 135 MW, and had a peak thermal flux of 4.0 × 10^14^ n/cm^2^/s^12^.

The NRU reactor core was comprised of many different types of rods, such as driver fuel rods, Mo-99 and Co-60 production rods, absorber rods, and control rods. The NRU driver fuel was a low-enriched uranium (LEU) fuel alloy of Al-61 wt% U_3_Si consisting of U_3_Si particles dispersed in a continuous aluminum matrix, with 19.8% U-235 in uranium.

### Neutron detectors

Two neutron detectors were employed in this work. One large-area neutron detector employed in this work was a boron-lined detector from Proportional Technologies, Inc. (Houston, TX, USA)^15^, which presented an active area of 1 × 0.18 m on its broadest sides. This detector, heretofore referred to as a Boron-Coated Straw (BCS) detector, consists of seven sealed aluminum tubes (2.54 cm diameter, 1 m long), each of which consists of 7 B-10 – enriched B_4_C coated straw detectors (each 7.5 mm in diameter, 1 m long). Each straw was filled with Ar/CO_2_ gas (90/10) at 10.5 psi. The detector had a total of 49 sealed straws. The straws were biased with a +1000 V high voltage supply. The 49 straws provided signal output at each end of the detector tubes, and these signal outputs were added together using a summing amplifier. A DC power supply provided ±5 V to each of the signal output ends, and the summing amplifier. The output of the summing amplifier was relayed via an Ortec 671 shaping amplifier, an Ortec 406 A single channel analyzer, and an Ortec 416 A gate and delay generator to a National Instruments (NI) cRIO-9023 real-time controller through a NI 9402 LVTTL high-speed bidirectional digital I/O module. The NI cRIO-9023 provided time-stamping of individual pulses. A detailed characterization of this detector using calibrated experimental measurements in comparison with simulations has been published elsewhere^16^.

The other large-area neutron detector consisted of seven sealed “B10+” stainless steel tubes (2.45 cm diameter, 101.6 cm active length) from General Electric Reuter Stokes (Twinsburg, OH, USA)^17^ lined with an elemental B-10 - enriched coating, and filled with 0.75 atm ^3^He, along with Ar and CO_2_, to a total pressure of 16.22 psi. The tubes collectively presented an active area of 1 × 0.18 m on the broadest sides, and were biased with +700 V. The high voltage was supplied by a NPM3100E neutron pulse monitor (NPM) from Quaesta Instruments (Tucson, AZ, USA), which also processed pulses through a charge sensitive amplifier, a fixed gain pulse-shaping amplifier, a variable gain amplifier, and an analog to digital converter, before using firmware algorithms to analyze the digitized data^18^. The NPM was used to record time-stamped pulses in list mode. A detailed characterization of this detector using calibrated experimental measurements in comparison with simulations has been published elsewhere^19^.

### Data acquisition method

For both neutron detectors, the acquired binary list mode files were off-line binned into a time-series histogram via C++ routines. The time-series plots provided a record of detector counts versus time, allowing one to examine changes in count rate that occur during measurement. The detector count rate as a function of time during the course of measurement was compared against NRU’s measured thermal reactor power as a function of time. The reactor thermal power was deduced from the total process water inlet flow minus the flow to facilities outside of the reactor core, such as fuel rod storage, air conditioning, and experimental loops; and the temperature difference between incoming and outgoing water flow.

## Data Availability

The data that support the findings of this study are available from the corresponding author upon reasonable request.

## References

[1] IAEA Department of Safeguards. *Safeguards: Staying Ahead of the Game* (IAEA Division of Public Information, Vienna, Austria, 2007).

[2] Boyer, B., & Schanfein, M. In *Nuclear Safeguards, Security, and Nonproliferation* (ed James, E. D.) (Elsevier, Oxford, UK, 2008).

[3] Anzelon, G. Antineutrino reactor monitoring in the context of IAEA safeguards. In *Workshop on Applied Antineutrino Physics (AAP 2018)* (Livermore, CA, USA, 2018).

[4] IAEA Department of Safeguards. *Long-Term R&D Plan, 2012–2023*, *STR-375*. (IAEA, Vienna, Austria, 2013).

[5] Prasad S, Abdulla A, Morgan MG, Azevedo IL (2015). Nonproliferation improvements and challenges presented by small modular reactors. Prog. Nucl. Energy.

[6] Ferguson, C. D., & Potter, W. C. In *Nuclear Safeguards, Security, and Nonproliferation* (ed James, E. D.) (Elsevier, Oxford, UK, 2008).

[7] Whitlock J, Sprinkle J (2012). Proliferation resistance considerations for remote small modular reactors. AECL Nucl. Rev..

[8] Beaumont JS, Mellor MP, Villa M, Joyce MJ (2015). High-intensity power-resolved radiation imaging of an operational nuclear reactor. Nat. Commun..

[9] Glasstone, S. & Sesonske, A. *Nuclear Reactor Engineering: Reactor Design Basics* 4th edn, Vol. 1, 103–106 (Springer Science+Business Media, Dordrecht, 1994).

[10] Hayes AC, Vogel P (2016). Reactor Neutrino Spectra. Annu. Rev. Nucl. Part. Sci..

[11] Madland, D. G. *New fission-neutron-spectrum representation for ENDF, LA-9285-MS*. (Los Alamos National Laboratory, Los Alamos, NM, USA, 1982).

[12] Leung, T. C. & Atfield, M. D. Validation of the TRIAD code used for the neutronic simulation of the NRU reactor. In *Canadian Nuclear Society - 30th Annual Canadian Nuclear Society Conference and 33rd CNS/SNA Student Conference 2009*, **2**, 839-849 (Canadian Nuclear Society, Toronto, ON, Canada, 2009).

[13] Harms N, Rodriguez P (1996). Safeguards at light-water reactors: Current practices, future directions. IAEA Bull..

[14] Levinsky, A. et al. Comparison of the main operational characteristics of the lead-cooled, gas-cooled and molten salt modular small modular reactor concepts. In *PHYSOR 2018:Reactor Physics Paving the Way Towards More Efficient Systems* (American Nuclear Society, La Grange Park, IL, USA, 2018).

[15] Proportional Technologies, Inc. https://proportionaltech.com/ (2019).

[16] van der Ende BM, Atanackovic J, Erlandson A, Bentoumi G (2016). Use of GEANT4 vs. MCNPX for the characterization of a boron-lined neutron detector. Nucl. Inst. Meth. Phys. Res. A.

[17] Baker Hughes a GE company. *Radiation Measurement* https://www.gemeasurement.com/radiation-measurement (2019).

[18] Quaesta Instruments. *Neuchrometer* https://www.quaestainstruments.com/neuchrometer (2019).

[19] van der Ende BM, Rand ET, Erlandson A, Li L (2018). Use of SRIM and Garfield with Geant4 for the characterization of a hybrid ^10^B/^3^He neutron detector. Nucl. Inst. Meth. Phys. Res. A.

[20] Mughabghab, S. F. *Atlas of Neutron Resonances, Resonance Parameters and Thermal Cross Sections, Z=1-100* 5th edn, (Elsevier, Amsterdam, 2006).

[21] James MF (1969). Energy released in fission. J. Nucl. Energy.

